# Hyponatremia, Inflammation at Admission, and Mortality in Hospitalized COVID-19 Patients: A Prospective Cohort Study

**DOI:** 10.3389/fmed.2021.748364

**Published:** 2021-12-02

**Authors:** Juan Carlos Ayus, Armando Luis Negri, Michael L. Moritz, Kyung Min Lee, Daniel Caputo, Maria Elena Borda, Alan S. Go, Carlos Eghi

**Affiliations:** ^1^Department of Nephrology, University of California, Irvine, Orange, CA, United States; ^2^Nephrology Section, Instituto de Investigaciones Metabólicas, Universidad del Salvador, Buenos Aires, Argentina; ^3^Children's Hospital of Pittsburgh, University of Pittsburgh Medical Center (UPMC), Pittsburgh, PA, United States; ^4^Division of Nephrology, Department of Pediatrics, The University of Pittsburgh School of Medicine, Pittsburgh, PA, United States; ^5^Epidemiology Division, Hospital Posadas, Buenos Aires, Argentina; ^6^Nephrology Section, Hospital Posadas, Buenos Aires, Argentina; ^7^Preventive Medicine Section, Hospital Posadas, Buenos Aires, Argentina; ^8^Division of Research, Kaiser Permanente Northern California, Oakland, CA, United States; ^9^Department of Health Systems Science, Kaiser Permanente Bernard J. Tyson School of Medicine, Pasadena, CA, United States; ^10^Departments of Medicine (Nephrology), Epidemiology and Biostatistics, University of California, San Francisco, San Francisco, CA, United States; ^11^Departments of Medicine (Nephrology), Health Research and Policy, Stanford University, Palo Alto, CA, United States

**Keywords:** hyponatremia, COVID-19, mortality, inflammation, C-reactive protein

## Abstract

**Background:** Systemic inflammation has been associated with severe coronavirus disease 2019 (COVID-19) disease and mortality. Hyponatremia can result from inflammation due to non-osmotic stimuli for vasopressin production.

**Methods:** We prospectively studied 799 patients hospitalized with COVID-19 between March 7 and November 7, 2020, at Hospital Posadas in Buenos Aires, Argentina in order to evaluate the association between hyponatremia, inflammation, and its impact on clinical outcomes. Admission biochemistries, high-sensitivity C-reactive protein (hsCRP), ferritin, patient demographics, and outcome data were recorded. Outcomes (within 30 days after symptoms) evaluated included ICU admission, mechanical ventilation, dialysis-requiring acute kidney injury (AKI), and in-hospital mortality. Length of hospital stay (in days) were evaluated using comprehensive data from the EHR.

**Results:** Hyponatremia (median Na = 133 mmol/L) was present on admission in 366 (45.8%). Hyponatremic patients had higher hsCRP (median 10.3 [IR 4.8–18.4] mg/dl vs. 6.6 [IR 1.6–14.0] mg/dl, *p* < 0.01) and ferritin levels (median 649 [IQR 492–1,168] ng/dl vs. 393 [IQR 156–1,440] ng/dl, *p* = 0.02) than normonatremic patients. Hyponatremia was associated with higher odds of an abnormal hsCRP (unadjusted OR 5.03, 95%CI: 2.52–10.03), and remained significant after adjustment for potential confounders (adjusted OR 4.70 [95%CI: 2.33–9.49], *p* < 0.01). Hyponatremic patients had increased mortality on unadjusted (HR 3.05, 95%CI: 2.14–4.34) and adjusted (HR 2.76, 95%CI:1.88–4.06) in Cox proportional hazard models. Crude 30-day survival was lower for patients with hyponatremia at admission (mean [SD] survival 22.1 [0.70] days) compared with patients who were normonatremic (mean [SD] survival 27.2 [0.40] days, *p* < 0.01).

**Conclusion:** Mild hyponatremia on admission is common, is associated with systemic inflammation and is an independent risk factor for hospital mortality.

**Clinical Trial Registration:** www.ClinicalTrials.gov, identifier NCT04493268.

## Introduction

The novel coronavirus disease 2019 (COVID-19) or SARS CoV-2 is the cause of an acute respiratory illness which has spread around the world. Coronavirus disease 2019 infected patients develop systemic inflammation (1). Uncontrolled inflammation leads to a cytokine storm (2), an extreme release of cytokines in response to infection, which causes acute respiratory distress syndrome with bilateral pneumonia and multiple organ failure (3–6).

Hyponatremia is the most common electrolyte disorder in hospitalized patients and occurs in about 30% of patients with pneumonia (7, 8). Hyponatremia can result from inflammation due to non-osmotic stimuli for vasopressin production (9) and is a risk factor for death in general hospitalized patients (10). Thus, hyponatremia linked to severe inflammation could be another prognostic factor for poor outcomes in COVID 19 infected patients as has been recently shown in a recent meta-analysis (11). However, all the previous studies have been retrospective or cross-sectional in nature. We found no other studies in the literature that have analyzed the prevalence and severity of hyponatremia among hospitalized for COVID-19 in a prospective way.

In the present study, we prospectively evaluated the prevalence and severity of hyponatremia among patients hospitalized for COVID-19, its association with inflammatory parameters, in-hospital clinical outcomes, and mortality.

## Methods

### Study Sample and Classification of Normonatremia and Hyponatremia Status

**Figure 1:**
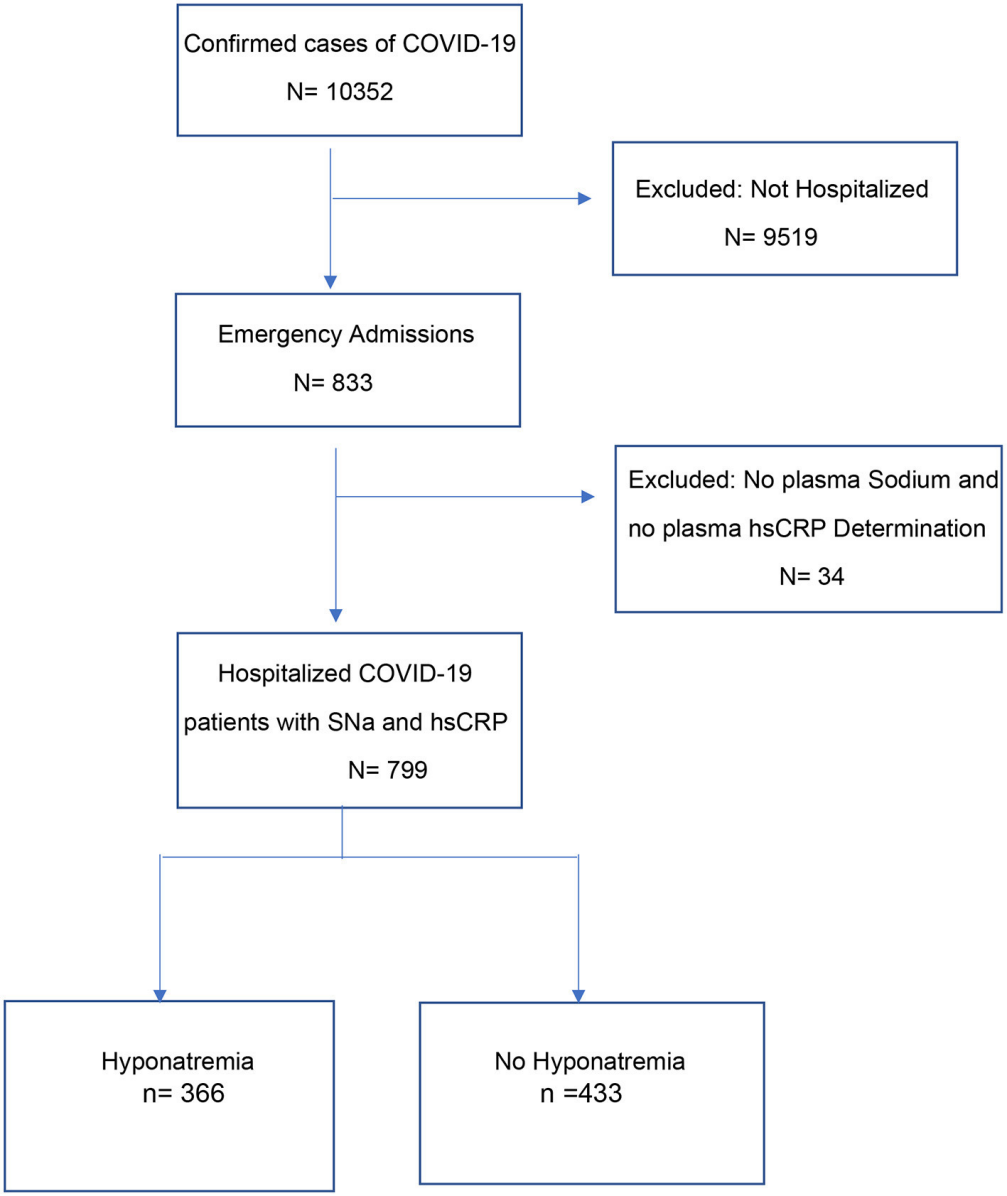
Cohort assembly of eligible patients hospitalized for COVID-19.

Between March 7 2020 and November 7 2020, 10,352 patients were diagnosed SARS-CoV-2 infection by PCR from nasopharyngeal swabs at the Posadas Hospital in Buenos Aires, Argentina. Eight hundred and thirty-three of the patients were hospitalized, of which 799 were included in the study (Figure 1). Patients were hospitalized if they were older (>60 years), had one or more comorbidities, were immunosuppressed, had uni- or bi-lateral pulmonary compromise or had oxygen desaturation (<95%).

Using a multivariate backward-staggered Cox regression model adjusted for age, morbid obesity, acute kidney injury (AKI), hypernatremia, non-dialysis-requiring chronic kidney disease (CKD), receiving maintenance dialysis, HIV, asthma, heart failure, chronic liver disease, cancer, and other comorbidities, we also evaluated the association of hyponatremia and all-cause in-hospital death at 30 days.

Of these patients, only those who had in their admission biochemistries serum sodium and high-sensitivity C-reactive protein (hsCRP) determined were included in this study.

All plasma sodium concentration measurements were performed using the ion-selective electrode method (normal range 136–145 mmol/L). Plasma sodium levels were corrected for plasma glucose using the following formula: corrected plasma sodium (mEq/L) = measured plasma sodium (mEq/L) + 0.016 – (plasma glucose [mg/dl] – 100). Using plasma sodium data available at hospital admission, patients were classified as hyponatremic if plasma sodium were ≤ 135 mmol/L. These patients were classified as having mild hyponatremia if serum sodium was between 131 and 135 mmol/L, moderate hyponatremia if sodium was between 126 and 130 mmol/L, or severe hyponatremia if sodium values were <126 mmol/L.

Data sources for the study included information extracted from the institution's electronic health record (EHR) system (including data from the central laboratory). Patient‘s demographics, clinical presentation characteristics, laboratory determinations, comorbidities, and outcome data were recorded. The study was approved by the institutional ethics committee and was carried out in accordance with the principles outlined in the Declaration of Helsinki. A waiver of informed consent was obtained due to the nature of the study (www.ClinicalTrials.gov Identifier: NCT04493268).

### Covariates

We used as covariates admission patient characteristics that could be associated with increased hospital mortality as older age (>65 years), male sex, hypertension, diabetes mellitus, obesity (BMI > 30), morbid obesity (BMI > 40), asthma, chronic obstructive pulmonary disease, previous pneumonia, former smoker, current smoker, hypernatremia, AKI, non-dialysis-requiring CKD (defined as an estimated glomerular filtration rate <60 ml/min/1.73 m^2^, using the equation from the Modification of Diet in Renal Disease Study), receiving maintenance dialysis, congestive heart failure, chronic liver disease, cancer, other immunodeficiency disorders, pulmonary tuberculosis, chronic neurological diseases, pregnancy, and the Charlson comorbidity score.

### Follow-Up and Outcomes

Follow-up occurred through November 7, 2020. We analyzed in-hospital outcomes (up to 30 days after symptom) that included length of hospital stay, admission to the ICU during hospitalization, mechanical ventilation use, Dialysis-requiring AKI, and in-hospital mortality.

### Admitted Patients for Different Pathologies Who Got Infected With SARS-CoV-2 in Hospital

Eighty-four patients who were admitted to the hospital for different pathologies and who got infected with SARS-CoV-2 were also included in the study.

### Statistical Analysis

We performed all analyses using SPSS version 21. A two-sided *p*-value < 0.05 value was considered significant. Categorical variables were described using frequencies and proportions and compared across groups using a chi-square or Fischer's exact test. Continuous variables were described using medians and interquartile ranges (IQR) and compared across groups using the Mann-Whitney U- or Kruskal-Wallis-test. Correlation studies and logistic regression models were performed that adjusted for age and gender to evaluate the association between hyponatremia and hsCRP level. The associations between hyponatremia and clinical outcomes (i.e., ICU admission, use of mechanical ventilation, in-hospital death) were assessed with unadjusted and multivariable logistic regression models that included variables associated with death using backward stepwise selection (retention *P*-value < 0.2). The association of hyponatremia and all-cause in-hospital death at 30 days was evaluated using the Kaplan Meier method with a log-rank test as well as unadjusted and multivariable Cox regression. The multivariable Cox regression model included variables associated with 30-day in-hospital mortality using backward stepwise selection (retention *P*-value < 0.2).

## Results

Among 799 hospitalized patients for COVID-19 infection, hyponatremia was present on admission in 366 (45.8%), with 310 (84.7%) of these patients having mild hyponatremia (sodium levels between 131 and 135 mEq/L) (Table 1). Hyponatremic patients were older and more frequently men, had higher frequency of pneumonia (radiological-clinical) and lower oxygen saturation.

**Table 1:** Demographic and clinical characteristics, laboratory values, and comorbidities in hospitalized patients with COVID-19 with and without hyponatremia on admission.

	**Serum sodium**	**Serum sodium**	***p*-Values**
	**≤135 mEq/L**	**>135 mEq/L**	
	***n* = 366**	***n* =433**	
**Demographic characteristics**			
Age, years	59.25 (48.53–66.60)	53.96 (41.68–65.37)	<0.01
Men	244 (66.7%)	242 (55.9%)	<0.01
**Presenting symptoms and vital signs**			
Anosmia	41 (11.3%)	68 (15.9%)	0.06
Arthralgia	16 (4.4%)	17 (4.0%)	0.75
Headaches	83 (23.0%)	85 (19.9%)	0.29
Coma	1 (0.3%)	3 (0.7%)	0.63
Confusion	9 (2.5%)	12 (2.8%)	0.78
Seizures	4 (1.1%)	3 (0.7%)	0.71
Diarrhea	50 (13.9%)	47 (11.0%)	0.23
Dysgeusia	42 (11.6%)	48 (11.2%)	0.86
Dyspnea	216 (59.8%)	240 (56.2%)	0.30
Abdominal pain	22 (6.1%)	15 (3.5%)	0.09
Chest pain	37 (10.2%)	43 (10.1%)	0.93
Conjunctivitis	1 (0.3%)	4 (0.9%)	0.38
General discomfort	122 (33.8%)	129 (30.2%)	0.28
Myalgia	75 (20.8%)	89 (20.9%)	0.97
Odynophagia	93 (25.8%)	125 (29.3%)	0.27
Cough	240 (66.5%)	282 (66.0%)	0.90
Vomit	34 (9.4%)	30 (7.0%)	0.22
Fever 37°5–37°9	5 (1.4%)	20 (4.7%)	<0.01
Fever >38°	244 (67.6%)	261 (61.1%)	0.06
Pneumonia (radiological-clinical)	18 (5.0%)	9 (2.1%)	0.03
Oxygen saturation (%)	93 (89–96)	94 (90–97)	<0.01
**Laboratory values**			
Serum Sodium (mEq/L)	133 (131.7–134.2)	138 (137–140)	<0.01
Category of hyponatremia			
Serum sodium (mEq/L) 131–135	310 (84.7%)	N/A	
Serum sodium (mEq/L) 126–130	45 (12.3%)	N/A	
Serum sodium (mEq/L) <126	11 (3.0%)	N/A	
Serum glucose (g/L)	1.17 (1.04–1.45)	1.28 (1.05–1.78)	<0.01
Blood leukocytes/mm^3^	7,850 (5,700–10,725)	7,250 (5,600–9,900)	0.26
Blood platelets/1,000 mm^3^	207 (159.75–265.25)	209.50 (168–268)	0.39
**Comorbidities**			
Hypertension	150 (41.2%)	128 (29.8%)	<0.01
Diabetes mellitus	89 (24.5%)	102 (23.7%)	0.81
Obesity	49 (13.5%)	74 (17.2%)	0.15
Morbid obesity	2 (0.5%)	11 (2.6%)	0.03
Asthma	13 (3.6%)	35 (8.1%)	<0.01
Chronic obstructive pulmonary disease	7 (1.9%)	14 (3.3%)	0.24
Previous pneumonia	7 (1.9%)	11 (2.6%)	0.55
Former smoker	38 (10.4%)	44 (10.2%)	0.92
Current smoker	13 (3.6%)	25 (5.8%)	0.14
Non-dialysis chronic kidney disease	20 (5.5%)	10 (2.3%)	0.02
Receiving maintenance dialysis	16 (4.4%)	12 (2.8%)	0.22
Congestive heart failure	22 (6.0%)	22 (5.1%)	0.57
Chronic liver disease	6 (1.6%)	1 (0.2%)	0.03
Cancer	33 (9.1%)	18 (4.2%)	<0.01
Other immunodeficiency disorder	18 (4.9%)	21 (4.9%)	0.97
HIV	4 (1.1%)	7 (1.6%)	0.52
Pulmonary TB	9 (2.5%)	7 (1.6%)	0.40
Chronic neurological diseases	28 (7.7%)	36 (8.4%)	0.73
Pregnancy	3 (0.8%)	6 (0.8%)	0.45
Acute kidney injury (AKI)	22 (6%)	23 (5.3%)	0.67
Charlson comorbidity index (CCI)	2 (1–4)	2 (0–3)	<0.01
**Risk factors associated with hyponatremia**			
Antihypertensive drugs	63 (17.2%)	61 (14.1%)	0.22
Antiepileptic drugs, antidepressant use, and others	10 (2.7%)	17 (3.9%)	0.35
Thiazides	0 (0%)	4 (0.9%)	0.13
Hypothyroidism	24 (6.6%)	18 (4.2%)	0.17

*Age, oxygen saturation, Laboratory values, and CCI: Median (IQR); presenting symptoms, vital signs, and comorbidities: N (%)*.

**Figure 2:**
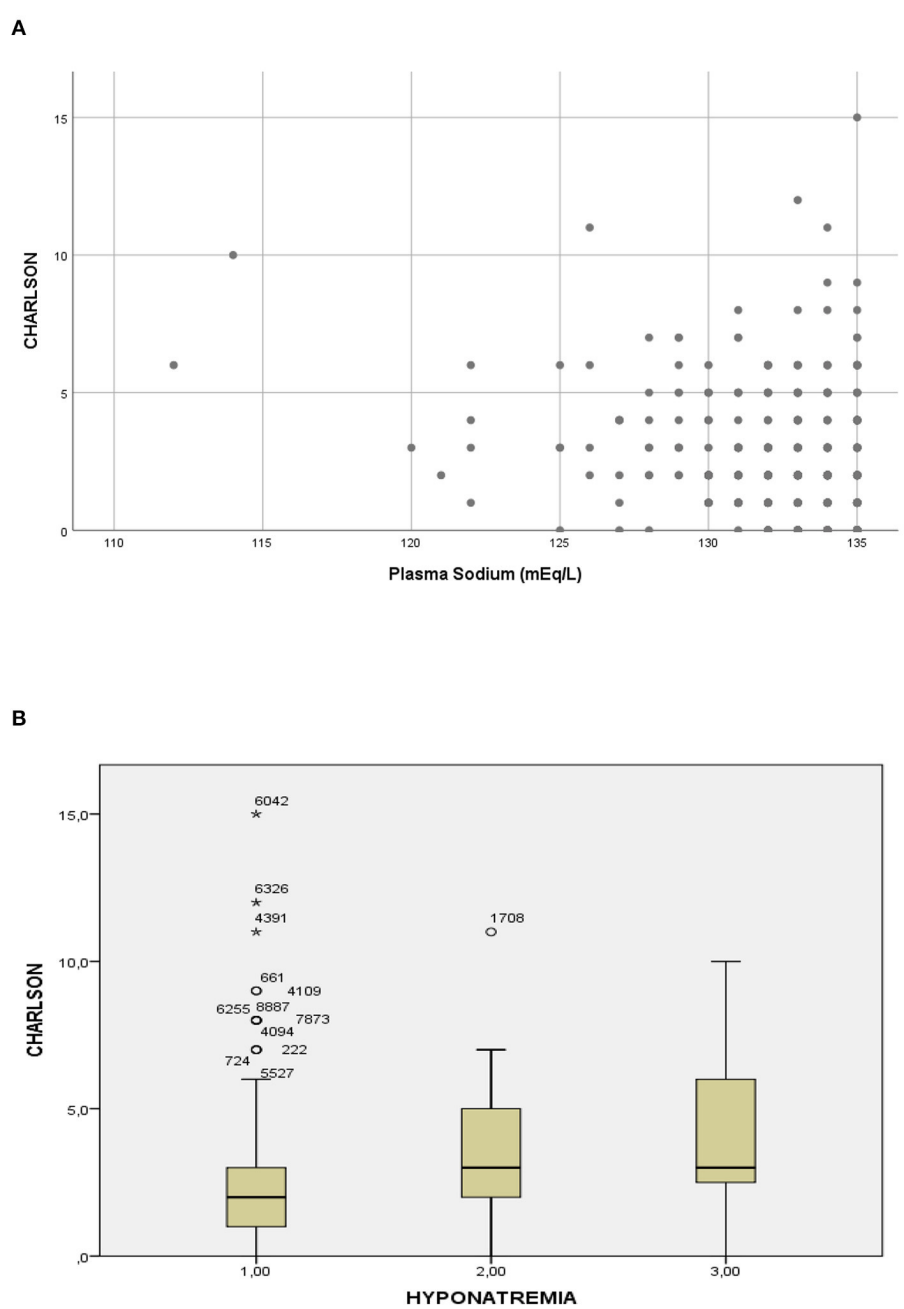
Relationship between plasma sodium and the Charlson comorbidity index. **(A)** Scatterplot of the Charlson comorbidity index according to the plasma sodium (Spearman test: rho = 0.21; *p* < 0.01). **(B)** Boxplot of the Charlson comorbidity index according to severity of hyponatremia, group 1 ≤ 126 mEql/L, group 2 = 126–130 mEq/L and group 3 = 131–135 Eq/L (Kruskal-Wallis test: *p* < 0.01).

Patients with hyponatremia had more frequently hypertension, non-dialysis-requiring CKD, chronic liver disease, and cancer (Table 1). Patients with the most severe hyponatremia had less comorbidities along with a Charlson comorbidity index significantly lower than patients in the intermediate and higher plasma sodium groups, with a significant correlation observed between the Charlson index and the plasma sodium (Figure 2).

### Hyponatremia and Inflammation Markers

Hyponatremic patients had higher hsCRP levels than normonatremic patients (median 10.3 [IQR 4.8–18.4] mg/dl vs. 6.6 [IQR 1.6–14.0] mg/dl, respectively, *p* < 0.01) (Table 2). Univariate logistic regression analyses showed that hyponatremia at admission was associated with higher odds of an abnormal hsCRP (unadjusted OR 5.03, 95%CI: 2.52–10.03). Multiple logistic regression analyses showed after adjustment for potential confounders that hyponatremia remained associated with higher hsCRP (adjusted OR 4.70 [95%CI: 2.33–9.49], *p* < 0.01).

**Table 2:** Inflammatory parameters in hospitalized patients with COVID-19 with and without hyponatremia on admission.

**Variable**	**Serum sodium**	**Serum sodium**	***p*-Values**
**Median (IQR)**	**≤135 mEq/L**	**>135 mEq/L**	
	***n* = 366**	***n* = 433**	
High-sensitivity CRP (mg/dl)	10.35 (4.80–18.40)	6.60 (1.60–14.00)	<0.01
High-sensitivity CRP >0.5 mg/dl^*^	356 (97.3 %)	379 (87.5%)	<0.01
Serum ferritin (ng/ml)	649 (492–1,168)	393 (156–1,440)	0.02
Serum d-dimer (ng/ml)	540 (397–789)	386 (241.5–791)	<0.01

* *N (%)*.

Hyponatremic patients had higher serum ferritin levels than normonatremic patients (median 649 [IQR 492–1,168] ng/dl vs. 393 [IQR 156–1,440] ng/dl, respectively, *p* = 0.02), they also had significantly higher serum d-dimer levels (Table 2).

### Hyponatremia and Outcomes

**Figure 3:**
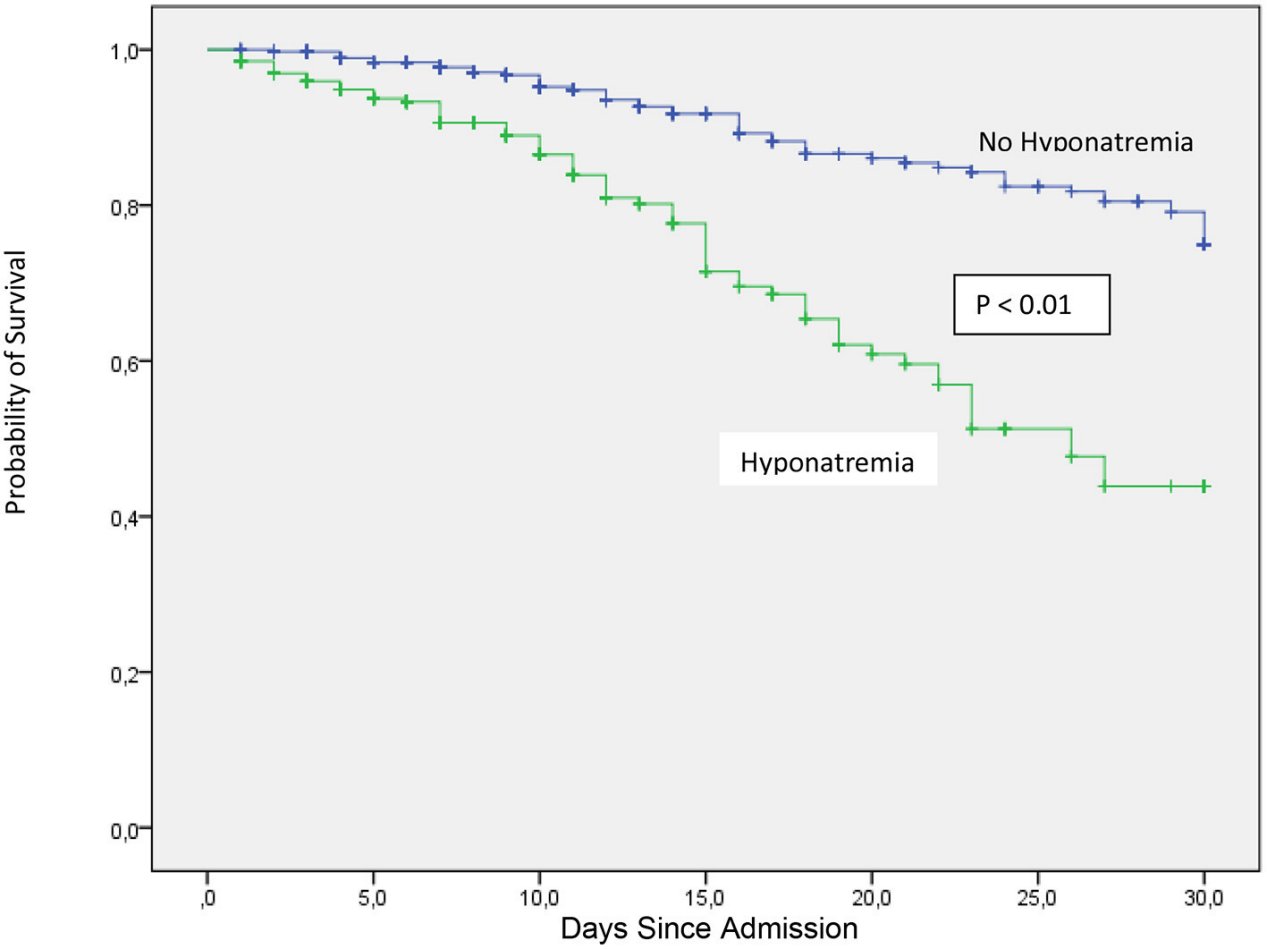
Thirty-day in-hospital mortality between COVID-19 patients at admission with or without hyponatremia.

Among in hospital outcomes, length of hospital stay and admission to the ICU during hospitalization were borderline significant between hyponatremic and non-hyponatremic patients (Table 3). In hospital mortality was higher for hyponatremic patients in an unadjusted model (OR 1.90, 95%CI: 1.30–2.78) and persisted significantly higher after adjustment for age, AKI, heart failure, receiving maintenance dialysis, chronic liver disease, chronic obstructive pulmonary disease, and cancer (OR 1.80, 95% CI:1.16–2.81). Crude 30-day survival was lower for patients with hyponatremia at admission (mean [SD] survival 22.1 [0.69] days) compared with patients who were non-hyponatremic (mean [SD] survival 27.2 [0.39] days, *p* < 0.01) (Figure 3).

**Table 3:** Clinical Outcomes in hospitalized patients with COVID-19 with and without hyponatremia on admission.

**Variable**	**Serum sodium**	**Serum sodium**	***p*-Values**
***N* (%)**	**≤135 mEq/L**	**>135 mEq/L**	
	***n* = 366**	***n* = 433**	
Length of stay (days)^*^	8 (4–15)	7 (3–13)	0.053
ICU admission during hospitalization	95 (26.5%)	87 (20.5%)	0.050
Dialysis-requiring AKI	3 (0.9%)	2 (0.5%)	0.67
Mechanical ventilation	70 (19.7%)	64 (15.5%)	0.12
In-hospital deaths	78 (21.3%)	54 (12.5%)	<0.01
Hyponatremia at the time of death	39 (50.6%)	12 (22.2%)	<0.01

* *Median (IQR)*.

**Figure 4:**
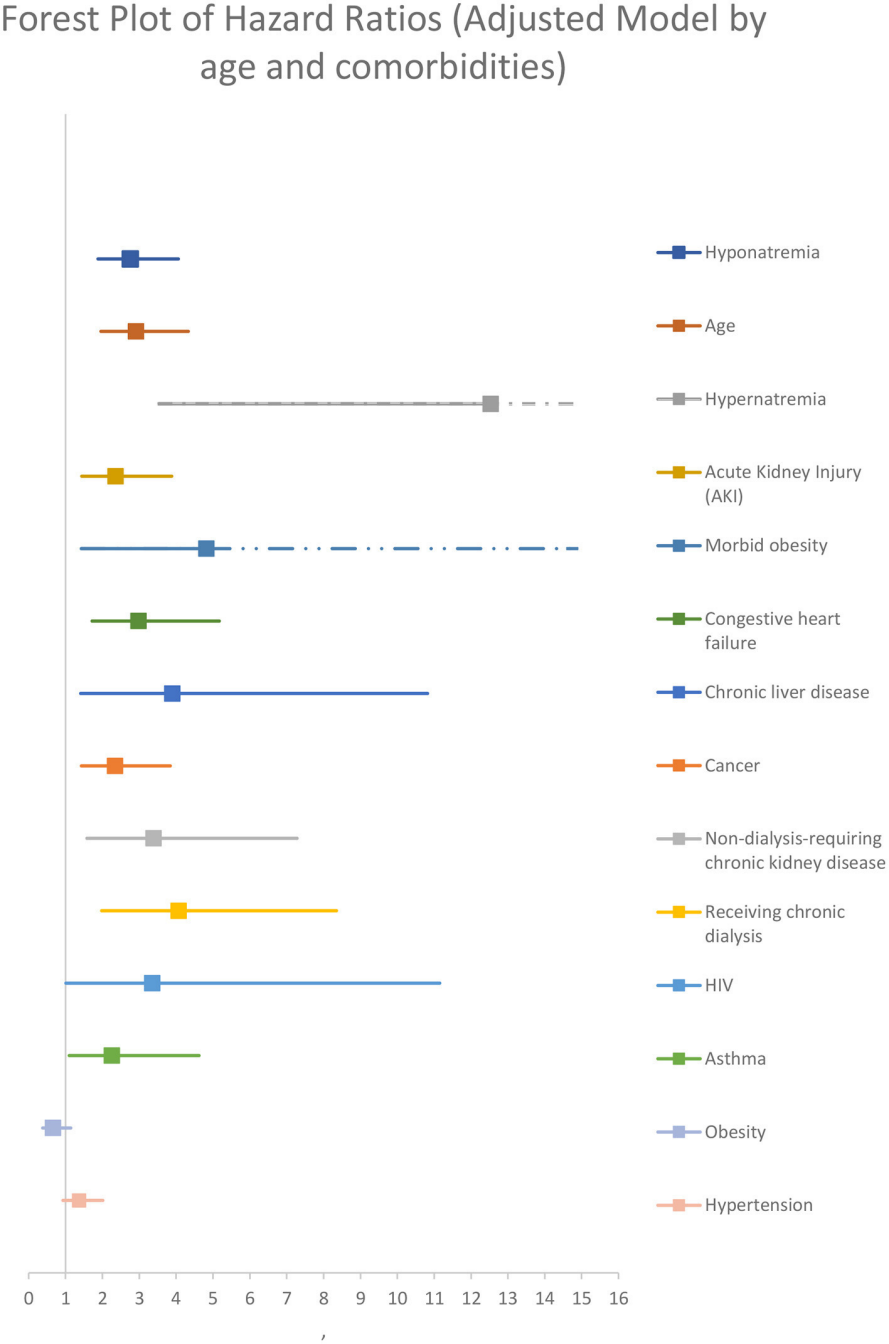
Adjusted model by age and comorbidities.

After performing a multivariate backward stepwise Cox regression model adjusted for age, hypernatremia, morbid obesity, AKI, non-dialysis-requiring CKD, receiving maintenance dialysis, HIV, asthma, heart failure, chronic liver disease, and cancer, hyponatremia at admission was associated with a higher rate of all-cause in-hospital mortality at 30 days (adjusted HR 2.76 [95% CI:1.88–4.06]) (Figure 4; Table 4).

**Table 4:** Relationship between hyponatremia and 30 days in-hospital death in hospitalized patients with COVID-19.

**Relationship between hyponatremia and 30 days in-hospital death**				
**Hazard ratio (95% confidence interval)**				
**Variable**	**Unadjusted model**	**Adjusted model by age and comorbidities**	**Adjusted model by Charlson score and comorbidities**	**Sensitivity analysis**
Hyponatremia	**3.05 (2.14-4.34)**	**2.76 (1.88-4.06)**	**2.51 (1.72–3.65)**	**3.08 (1.35–6.99)**
Age	–	**2.91 (1.96–4.33)**	–	**2.45 (1.1–5.43)**
Hypernatremia	–	**12.53 (3.54–44.35)**	**6.69 (1.94–23.09)**	–
Acute kidney injury (AKI)	–	**2.36 (1.44–3.88)**	**3.13 (1.96–5.0)**	1.71 (0.67–4.36)
Morbid obesity	–	**4.82 (1.43–16.2)**	**3.38 (1.02–11.22)**	–
Congestive heart failure	–	**2.98 (1.72–5.17)**	–	–
Chronic liver disease	–	**3.9 (1.41–10.82)**	–	–
Cancer	–	**2.34 (1.43–3.84)**	–	–
Non-dialysis-requiring chronic kidney disease	–	**3.39 (1.58–7.28)**	–	0.65 (0.18–2.36)
Receiving chronic dialysis	–	**4.07 (1.98–8.35)**	–	–
HIV	–	**3.35 (1.01–11.15)**	–	–
Asthma	–	**2.26 (1.1–4.62)**	1.81 (0.91–3.62)	–
Obesity	–	0.66 (0.38–1.14)	0.66 (0.39–1.12)	0.35 (0.08–1.53)
Chronic neurological diseases	–	–	–	**2.38 (1.02–5.58)**
Hypertension	–	1.37 (0.93–2.01)	1.4 (0.96–2.03)	0.69 (0.3–1.58)
Charlson score	–	–	**1.31 (1.23–1.4)**	**–**

*p-Values < 0.05 are highlighted in bold*.

**Figure 5:**
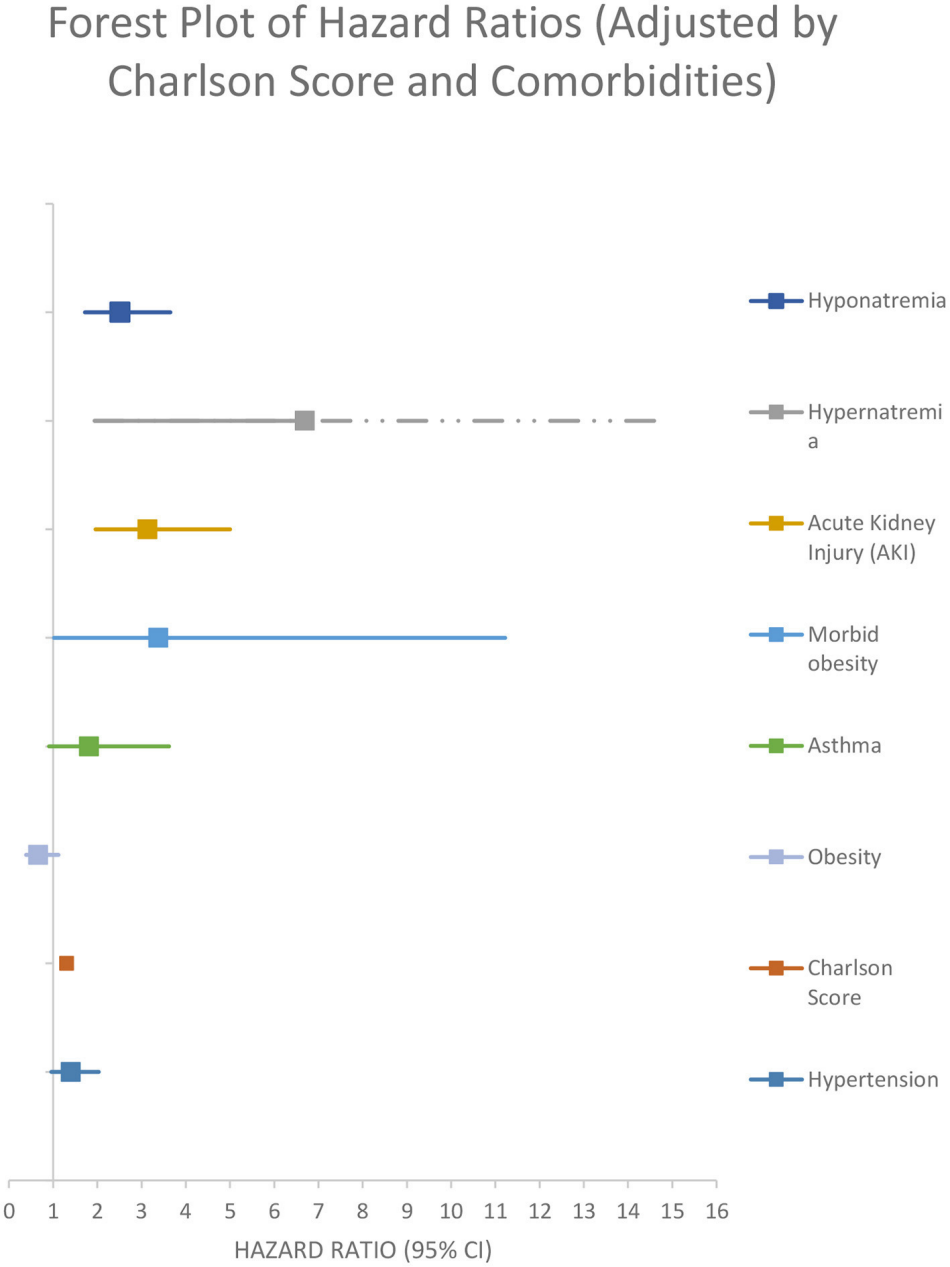
Adjusted model by Charlson score and comorbidities.

After performing a multivariate backward stepped Cox regression model adjusted for hypernatremia, morbid obesity, AKI, and Charlson score (the variables included in the Charlson score were not reintroduced so as not to generate collinearity), hyponatremia at the time of admission was associated with a higher rate all cause 30-day in hospital mortality for (adjusted HR 2.51 [95% CI: 1.72–3.65]) (Figure 5; Table 4).

**Figure 6:**
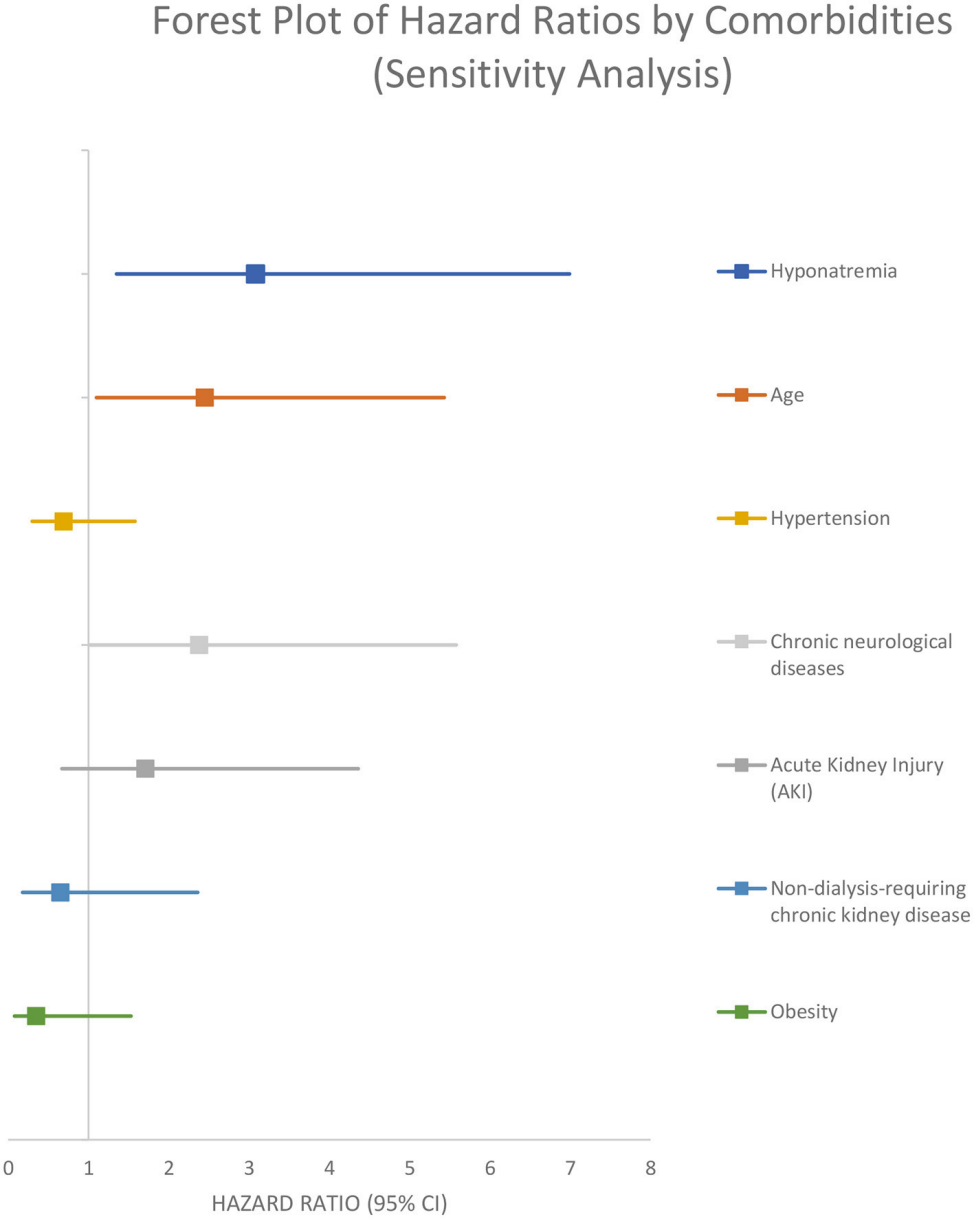
Sensitivity analysis.

In the sensitivity analysis performed on the 84 patients that were admitted to the hospital for different pathologies and got infected with SARS-CoV-2, the presence of hyponatremia was associated with a higher rate of 30 days in-hospital mortality from all causes (adjusted HR 3.08 [95% CI 1.35–6.99]) (Figure 6; Table 4).

## Discussion

In this prospective study of 799 patients with COVID 19, we found that 45.8% of them had mild hyponatremia on admission (serum Na between 131 and 135 mEq/L). Moreover, hyponatremic patients had higher hsCRP and serum ferritin levels than normonatremic patients (Table 2).

Univariate logistic regression analyses showed that hyponatremia at admission was associated with higher odds of an abnormal hsCRP (unadjusted OR 5.03, 95%CI: 2.52–10.03). Multiple logistic regression analyses showed after adjustment for potential confounders that hyponatremia remained associated with higher hsCRP (adjusted OR 4.70 [95%CI: 2.33–9.49], *p* < 0.01).

In-hospital mortality was higher for hyponatremic patients in an unadjusted model (OR 1.90, 95%CI: 1.30–2.78). Hyponatremia persisted as an independent risk factor for increased in hospital mortality after adjustment for age, AKI, and several comorbidities (OR 1.80, 95% CI: 1.16–2.81). Crude 30-day survival was lower for patients with hyponatremia at admission compared with normonatremic patients (Figure 3). Multivariable Cox regression survival analysis for in-hospital mortality adjusted for age, AKI, hypernatremia, and several comorbidities indicated a HR of 2.76 [95% CI:1.87–4.06] in hyponatremic patients (Figure 4; Table 4). In another model adjusted for hypertension and Charlson score, hyponatremia at the time of admission was associated with HR of 2.50 [95% CI: 1.72–3.65]) for in-hospital mortality (Figure 5; Table 4). A sensitivity analysis of 84 patients who were admitted to the hospital for different pathologies and who got infected with COVID 19 showed the same development of hyponatremia and inflammation as patients admitted with COVID 19 alone (Figure 6; Table 4). Thus, collectively, the present study suggests that mild hyponatremia at admission is associated with systemic inflammation and is an independent risk factor for hospital mortality.

The prevalence of hyponatremia at admission in patients with COVID 19 has been variable in different retrospective or cross-sectional series ranging from 30% (12, 13) to 47.8% (14). In our series, prevalence of hyponatremia was high (45.8%), probably because of the prospective nature of the study.

Hypovolemic hyponatremia is common in COVID 19 infected patients after admission because of the presence of increased insensible loses (taquipnea, fever), vomiting, diarrhea, and anorexia that accompany severe respiratory symptoms and the use of hypotonic fluids (11). However, in our population hyponatremia was present at admission before any therapeutic intervention took place. At admission no differences were found in vomiting, diarrhea, or fever between hyponatremic and non-hyponatremic patients (Table 1). Increased ADH release may be due non-osmotic ADH release associated with inflammation (9). IL-6 has been demonstrated as stimulus for ADH secretion from magnocellular neurons present in the hypothalamus (9). IL-6 is increased in COVID 19 infected patients with severe pneumonia and these higher IL-6 levels have been correlated with lower serum sodium levels in these patients (15). Our data supports that hyponatremia at admission is due to non-osmotic release of ADH as our hyponatremic patients had higher hsCRP and serum ferritin levels than non-hyponatremic patients (Table 2). Moreover, in recent paper by Duan et al. (16) the two factors with the best distinguishing power of progression to severe cases in patients with COVID 19 at admission were serum sodium and C-reactive protein (CRP) (15). Taken together these results suggest that inflammation is the main contributor to the hyponatremia seen at admission in our population.

Although several variables at admission have been associated with progression of COVID 19 cases to severe disease (15, 16) and with mortality during hospitalization (12), low serum sodium is an easily obtained electrolyte determination that can give early important prognostic information.

Although community-acquired pneumonia (CAP) is mainly caused by bacteria, viruses are a relevant etiologic factor for CAP. The main viral pathogens causing CAP are rhinovirus, respiratory syncytial virus, influenza viruses, parainfluenza viruses, and adenoviruses (17). Community-acquired pneumonia is one of the complications of influenza A (H1N1) infection and during the 2009 pandemic it was associated with high morbidity, admissions in ICU and mortality (18). In these patients, hyponatremia was independently associated with disease severity and was found in 17.6% of those with viral pneumonia. Hyponatremia at admission was also associated with longer hospital stay (19). In patients with avian-origin influenza (H7N9) almost half of the patients were hyponatremic (20).

Other viral infections associated with hyponatremia include human herpesvirus-6 (HHV-6), severe fever with thrombocytopenia syndrome virus (SFTSV), hantavirus, herpes simplex virus (HSV), and Ebola virus infections (21–23). Severe fever with thrombocytopenia syndrome virus infections are uncommon but are associated with high mortality. Hyponatremia was found to be an independent risk factor for death in SFTSV patients (22). In the non-survivor group, hyponatremia occurred in 60% of the cases while in the survivor group it was present in 20% of them. In Dengue infection, a frequent viral infection in Argentina, we found hyponatremia in 30.8% of 146 infected patients (24).

The strength of our study is its prospective design and the fact that the independent association of Hyponatremia with all cause 30 day in-hospital mortality persisted after adjustments in several models by age, several comorbidities, and Charlson Score. In sensitivity analysis performed on 84 patients that were admitted to the hospital for different pathologies and got infected with SARS-CoV-2 during hospital stay, the presence of hyponatremia was also associated with a higher rate of 30 day in-hospital mortality from all causes with a similar adjusted HR of 3.08 [95% CI: 1.35–6.99]). Thus, our prospective study confirms previous retrospective and cross-sectional studies that have shown a strong association of hyponatremia and mortality in COVID 19 infected patients.

The present study although have limitations. As an observational study, this study is susceptible to residual confounding, as it relied on information from electronic medical record to identified potential confounders and did not included all possible characteristics such as the use of certain counter medications (NSAIDs, proton pump inhibitors), that may be associated with the development of hyponatremia but were not systemically available. In addition, results from the study sample may not be fully generalizable to all population, given the limited racial/ethnic diversities. Thus, future studies are needed in other populations to confirm our findings.

In summary, this study found that mild hyponatremia at hospital admission is common finding in COVID 19 infected patients, is associated with systemic inflammation and is an independent predictor of mortality (2.76 increase vs. no hyponatremia).

## Data Availability Statement

The raw data supporting the conclusions of this article will be made available by the authors, without undue reservation.

## Ethics Statement

The studies involving human participants were reviewed and approved by Comite de Etica en Investigacion Hospital Nacional Prof. Alejandro Posadas. Written informed consent for participation was not required for this study in accordance with the national legislation and the institutional requirements.

## Author Contributions

JCA, CE, and AN contributed to conception and design of the study. CE and DC organized the data base. CE, KL, and MB performed the statistical analysis. JCA and AN wrote the first draft of the manuscript. All authors contributed to manuscript revision, read, and approved the submitted version.

## Conflict of Interest

The authors declare that the research was conducted in the absence of any commercial or financial relationships that could be construed as a potential conflict of interest.

## Publisher's Note

All claims expressed in this article are solely those of the authors and do not necessarily represent those of their affiliated organizations, or those of the publisher, the editors and the reviewers. Any product that may be evaluated in this article, or claim that may be made by its manufacturer, is not guaranteed or endorsed by the publisher.
